# Sensible Advice

**Published:** 1863-05

**Authors:** 


SENSIBLE ADVICE.—Dr. Miner, editor of the Buffalo Medical and Surgical Journal, addressed the graduates of the Buffalo Medical College as follows:

"He desired to do this for the benefit of the young men who are just entering the field of medical knowledge, that they may not neglect the longest lever of professional power, by which alone they may move the medical world. Medical colleges and their professors are a great power, and wield a wide influence, especially over medical students, and thus also over medical men. We are taught by them, however, in our pupilage the established and universally acknowledged principles of medicine, surgery, and the collateral sciences, while in actual practice we consult our written guides; then it is that those only who write are those who teach. Standard medical books are also a great power in the profession, but they are also dependent in great degree upon the Periodical Medical Press for both the material of which they are composed, and their adoption by the profession. Again, many physicians do not carefully read the voluminous works in medicine, while it is believed that all are more or less acquainted with periodical medical literature. These suggestions are made that this graduating class may not be unmindful of the importance to them of medical journals.

You may be of value to the journals, but the journals are of vastly greater value to you. They are practically the only available medium of communication with the profession; through them you will learn much of what you will know, and whatever of any professional importance is ever known of you, will be through such medium. Your attention is therefore called to the periodical medical press, as the all-powerful influence of the profession."—*Am. Medical Times.*

